# The importance of obstructive sleep apnea screening in driving license application procedures

**DOI:** 10.5249/jivr.v8i2.864

**Published:** 2016-07

**Authors:** Habibolah Khazaie, Mohammad Rasoul Ghadami, Maryam Masoudi

**Affiliations:** ^a^Sleep Disorders Research Center, Kermanshah University of Medical Sciences, Kermanshah, Iran.

There are numerous medical disorders that are known to heighten the risk of traffic collision and put drivers in serious danger. This important subject has persuaded governments to make legal solutions to deal with drivers who have these disorders. In order to acquire or renew a driving license, drivers must prove that they have no serious medical disorders which could impair their driving ability, or that they have taken the sufficient precautions to ensure adequate driving ability.

Obstructive sleep apnea (OSA) is one of these medical disorders and is related to excessive daytime sleepiness (EDS).^1^ It is a curable condition that can be successfully treated using continuous positive airway pressure (CPAP).^2^ OSA is an important risk factor for traffic collision.^3^ However, only a few countries, mostly in the European Union, require OSA screening prior to receiving or renewing a driving license.^4^

Despite the fact that the diagnosis of OSA and its dangerous consequences for driving have an important role in increasing safety on the road, screening for it is still not common practice. One significant problem is that OSA in drivers is generally evaluated by questionnaires, which are susceptible to reporting bias, because drivers usually seek to underestimate their OSA severity.^5^ However, the problem can be easily solved as objective evaluation by polysomnography or portable monitoring device at sleep clinics can confirm the diagnosis of OSA and define its severity. ^3,6^

It is very important to add the mandatory screening for OSA to the driving license application procedure, in which drivers who are suspected of having OSA must be referred to authorized sleep clinics for further evaluations before a driving license is issued. Suspect OSA sufferers must be advised not to drive until the OSA screening results are known. Drivers with moderate or severe OSA must receive appropriate treatment under medical insurance coverage. Licenses could be issued to drivers with OSA who have sufficient control of their condition and whose symptoms improve with appropriate treatment. This treatment must be administered by authorized sleep clinics. Drivers receiving treatment for OSA must be evaluated at regular intervals to determine the level of compliance with the treatment and its necessary continuation.
